# Supplementary material for: Tempo-spatial variations of zooplankton communities in relation to environmental factors and the ecological implications: A case study in the hinterland of the Three Gorges Reservoir area, China
Source: PLoS One. 2021 Aug 18;16(8):e0256313. doi: 10.1371/journal.pone.0256313 (PMC8372925; doi:10.1371/journal.pone.0256313)
Supplement: S1 Table — “+” denoted for the occurrence of species. (DOCX) [file pone.0256313.s001.docx]

## S1 Table The species composition of zooplankton during the three seasons. “+” denoted for the occurrence of species.

| NO. | Species | April 2018 | | | | | | | | | | | | August 2018 | | | | | | | | | | | | January 2019 | | | | | | | | | | | |
| --- | --- | --- | --- | --- | --- | --- | --- | --- | --- | --- | --- | --- | --- | --- | --- | --- | --- | --- | --- | --- | --- | --- | --- | --- | --- | --- | --- | --- | --- | --- | --- | --- | --- | --- | --- | --- | --- |
|  |  | Y1 | Y2 | Y3 | Y4 | Y5 | ZX1 | ZX2 | ZX3 | LB1 | LB2 | WQ1 | WQ2 | Y1 | Y2 | Y3 | Y4 | Y5 | ZX1 | ZX2 | ZX3 | LB1 | LB2 | WQ1 | WQ2 | Y1 | Y2 | Y3 | Y4 | Y5 | ZX1 | ZX2 | ZX3 | LB1 | LB2 | WQ1 | WQ2 |
| **Protozoans** | | | | | | | | | | | | | | | | | | | | | | | | | | | | | | | | | | | | | |
| 1 | *Arcella* sp. |  |  |  |  |  |  |  |  |  |  |  |  |  |  |  |  |  |  |  |  |  |  |  | **+** |  |  |  |  |  |  |  |  |  |  |  |  |
| 2 | *Arcella vulgaris* |  |  |  |  |  |  |  |  |  |  |  |  |  |  |  |  |  | **+** | **+** |  |  | **+** |  |  |  |  |  | **+** |  |  |  |  | **+** |  |  |  |
| 3 | *Arcella gibbosa* |  |  |  |  |  |  |  |  |  |  |  |  |  |  |  |  |  |  |  |  |  |  | **+** |  |  |  |  |  |  |  |  |  |  |  |  |  |
| 4 | *Centropyxis aculeata* |  |  |  |  |  |  | **+** |  | **+** |  |  |  |  |  | **+** |  | **+** |  |  |  |  |  | **+** |  |  |  |  |  |  |  |  | **+** |  |  |  | **+** |
| 5 | *Centropyxis ecornis* |  |  |  |  |  |  | **+** |  |  |  |  |  |  |  |  | **+** |  |  |  |  |  |  | **+** | **+** |  |  |  | **+** |  |  |  | **+** | **+** |  | **+** | **+** |
| 6 | *Difflugia urceolata* |  |  |  |  |  |  |  |  |  |  |  |  |  |  |  |  |  |  |  |  |  |  |  |  |  |  |  |  |  |  |  |  |  |  |  | **+** |
| 7 | *Difflugia globulosa* |  |  |  |  |  |  | **+** |  |  |  |  |  |  |  |  |  |  |  |  |  |  |  | **+** | **+** |  |  |  |  |  |  |  |  |  |  |  |  |
| 8 | *Difflugia acuminata* |  |  |  |  |  |  |  |  |  |  |  |  |  | **+** |  |  |  |  |  | **+** |  |  | **+** | **+** |  |  |  |  |  |  |  |  |  |  |  |  |
| 9 | *Difflugia corona* |  |  |  |  |  |  |  |  | **+** |  |  |  |  |  |  |  |  |  |  |  |  |  |  |  |  |  |  |  |  |  |  |  |  |  |  |  |
| 10 | *Difflugia oblonga* |  |  |  |  |  | **+** |  |  | **+** |  |  |  |  |  |  |  |  |  |  |  |  |  | **+** | **+** |  |  |  |  |  |  |  |  |  |  |  |  |
| 11 | *Euglypha rotunda* |  |  |  |  |  |  |  |  |  |  |  |  |  |  |  |  |  |  |  |  |  |  |  |  |  |  |  |  |  |  |  | **+** |  |  |  |  |
| 12 | *Acanthocystis aculeata* |  |  |  |  |  |  |  | **+** |  |  |  |  |  | **+** |  |  |  |  |  | **+** |  |  |  |  |  |  |  |  |  |  |  |  |  |  |  |  |
| 13 | *Lacrymaria olor* |  |  |  |  | **+** |  |  |  |  |  |  |  |  | **+** |  |  |  |  |  | **+** |  |  |  |  |  |  |  |  |  |  |  |  |  |  |  |  |
| 14 | *Didinium nasutum* |  |  |  |  |  |  |  |  |  |  |  |  |  |  |  |  |  |  |  | **+** |  |  |  |  |  |  |  |  |  |  |  |  |  |  |  |  |
| 15 | *Askenasia volvox* |  |  |  |  |  |  |  | **+** |  |  |  |  |  |  |  |  |  |  | **+** |  |  |  |  |  |  |  |  |  |  |  |  |  |  |  |  |  |
| 16 | *Actinobolina* sp. |  |  |  |  |  |  |  |  |  |  |  |  |  |  |  |  |  | **+** |  |  |  |  | **+** | **+** |  |  |  |  |  |  |  |  |  |  |  |  |
| 17 | *Litonotus* sp. |  |  |  |  |  |  |  |  |  |  | **+** |  |  |  |  |  |  |  |  |  |  |  |  |  |  |  |  |  |  |  |  |  |  |  |  |  |
| 18 | *Litonotus fasciola* |  |  |  |  |  |  |  |  |  |  | **+** |  |  |  |  |  |  |  |  |  |  |  |  |  |  |  |  |  |  |  |  |  | **+** |  |  |  |
| 19 | *Colpoda cucullus* |  |  |  |  |  |  |  |  |  |  |  |  |  |  |  |  |  |  |  |  |  |  |  |  |  |  |  |  |  |  |  |  | **+** |  |  |  |
| 20 | *Nassula* sp. |  |  |  |  |  |  |  |  |  |  |  |  |  |  |  |  |  |  |  |  |  |  |  |  |  |  |  |  |  |  |  |  | **+** |  |  |  |
| 21 | *Chilodonella* sp. |  |  |  |  |  |  | **+** |  |  | **+** | **+** | **+** |  |  |  |  |  |  |  |  |  |  |  |  |  |  |  |  |  |  |  |  | **+** |  |  |  |
| 22 | *Tetrahymena pyriformis* |  |  |  |  |  |  | **+** | **+** | **+** |  | **+** |  |  |  |  |  |  |  | **+** |  |  | **+** |  |  |  |  |  |  |  |  |  |  |  |  |  |  |
| 23 | *Glaucoma* sp. |  |  |  |  |  |  |  |  |  |  |  |  |  |  |  |  |  |  |  |  |  |  |  |  |  |  |  |  |  |  |  |  | **+** |  |  |  |
| 24 | *Paramecium* sp. |  |  |  |  |  |  |  |  |  | **+** |  |  |  |  |  |  |  | **+** | **+** |  | **+** | **+** |  |  |  |  |  |  |  |  |  |  |  |  |  |  |
| 25 | *Paramecium aurelia* |  |  |  |  |  |  |  |  |  |  |  |  |  |  |  |  |  |  |  |  |  |  |  |  |  |  |  |  |  |  |  | **+** | **+** |  | **+** |  |
| 26 | *Paramecium bursaria* |  |  |  |  |  |  |  |  | **+** |  | **+** | **+** |  |  |  |  |  |  |  |  |  |  |  |  |  |  |  |  |  |  |  |  | **+** |  |  |  |
| 27 | *Cyclidium* sp. |  |  |  |  |  |  |  |  |  | **+** |  |  |  |  |  |  |  | **+** | **+** |  |  |  |  | **+** |  |  |  |  |  |  |  |  |  |  |  |  |
| 28 | *Vorticella* sp. | **+** | **+** |  |  |  | **+** | **+** | **+** | **+** | **+** | **+** | **+** | **+** | **+** | **+** |  | **+** | **+** | **+** | **+** | **+** | **+** |  | **+** |  |  |  | **+** |  |  |  |  | **+** |  |  |  |
| 29 | *Vorticella elongata* |  |  |  |  |  |  |  |  |  |  |  |  |  |  |  |  |  |  |  |  |  | **+** |  |  |  |  |  |  |  |  |  |  |  |  |  |  |
| 30 | *Epistylis rotans* | **+** |  | **+** |  |  |  |  | **+** | **+** | **+** | **+** |  |  |  |  |  |  |  | **+** | **+** |  | **+** |  |  |  |  |  |  |  |  |  |  |  |  |  | **+** |
| 31 | *Tintinnopsis wangi* |  |  |  |  |  |  | **+** |  |  |  |  |  |  |  |  |  |  |  |  |  |  |  |  |  |  |  |  |  |  |  |  | **+** |  |  |  |  |
| 32 | *Tintinnopsis kiangsuensis* |  |  |  |  |  |  | **+** |  |  |  |  |  |  |  |  |  |  |  |  |  |  | **+** |  |  |  |  |  |  |  |  |  |  |  |  |  |  |
| 33 | *Tintinnopsis sinensis* |  |  |  |  |  |  | **+** |  |  |  |  |  |  |  |  |  |  |  |  |  |  |  |  |  |  |  |  |  | **+** |  |  |  |  |  |  |  |
| 34 | *Euplotes eurystomus* |  |  |  |  |  |  |  |  |  |  | **+** | **+** |  |  |  |  |  |  |  |  |  |  |  |  |  |  |  |  |  |  |  |  |  |  |  |  |
| 35 | Ciliate | **+** |  | **+** |  |  | **+** | **+** | **+** | **+** | **+** |  | **+** | **+** | **+** | **+** |  | **+** |  | **+** | **+** | **+** | **+** | **+** | **+** |  |  |  |  |  |  |  |  |  |  | **+** |  |
| Rotifers | | | | | | | | | | | | | | | | | | | | | | | | | | | | | | | | | | | | | |
| 36 | *Habrotrocha* sp. |  |  |  |  |  |  |  |  |  |  |  |  |  |  |  |  |  | **+** | **+** | **+** |  |  | **+** | **+** |  |  |  |  |  |  |  |  |  |  |  |  |
| 37 | *Rotaria tardigrada* |  |  |  |  |  |  |  |  |  |  |  |  |  |  | **+** |  |  |  |  | **+** |  |  |  |  |  |  |  |  |  |  |  |  |  |  |  |  |
| 38 | *Rotaria neptunia* |  |  |  |  |  |  |  |  |  |  |  |  |  |  |  |  |  |  | **+** |  |  |  |  |  |  |  |  |  |  |  |  |  |  |  |  | **+** |
| 39 | *Philodina* *erythrophthalma* |  |  |  |  |  |  |  |  |  |  |  |  |  |  |  |  |  |  | **+** | **+** |  | **+** |  | **+** |  | **+** |  |  | **+** |  |  |  | **+** |  | **+** | **+** |
| 40 | *Anuraeopsis coelata* |  |  |  |  |  | **+** |  | **+** |  |  |  |  |  |  |  |  |  |  | **+** | **+** |  |  | **+** |  |  |  |  |  |  |  |  |  |  |  |  |  |
| 41 | *Anuraeopsis fissa* |  |  |  |  |  |  | **+** | **+** |  |  |  | **+** |  |  |  |  |  | **+** | **+** | **+** | **+** | **+** |  | **+** |  |  |  |  |  |  |  |  |  |  |  |  |
| 42 | *Brachionus angularis* | **+** |  |  |  |  |  | **+** | **+** |  |  |  |  |  |  |  |  |  |  |  |  |  |  |  |  |  |  |  |  |  |  |  |  | **+** |  |  | **+** |
| 43 | *Brachionus calyciflorus* |  |  |  |  |  | **+** | **+** |  |  |  | **+** |  |  | **+** |  |  |  | **+** | **+** | **+** |  |  |  | **+** |  |  |  |  |  |  |  |  | **+** |  |  |  |
| 44 | *Brachionus diversicornis* | **+** |  |  |  |  |  |  | **+** |  |  |  |  |  | **+** |  |  |  | **+** | **+** | **+** |  | **+** |  |  |  |  |  |  |  |  |  |  |  |  | **+** |  |
| 45 | *Brachionus forficula* |  |  |  |  |  |  |  |  |  |  |  |  |  |  |  |  |  | **+** |  |  |  |  |  | **+** |  |  |  |  |  |  |  |  |  |  |  |  |
| 46 | *Brachionus leydigi* |  |  | **+** |  |  | **+** |  | **+** |  |  |  |  |  |  |  |  |  |  |  |  |  |  |  |  |  |  |  |  |  |  |  |  |  |  |  |  |
| 47 | *Brachionus nilsoni* |  |  |  |  |  |  |  |  |  |  |  |  |  |  |  |  |  |  |  |  |  |  |  |  |  |  |  |  |  |  |  |  | **+** |  | **+** |  |
| 48 | *Brachionus quadridentatus cluniorbicularis* | |  |  |  |  |  |  |  |  |  |  |  |  |  | **+** |  |  | **+** | **+** | **+** |  |  |  | **+** |  |  |  |  |  |  |  |  |  |  |  |  |
| 49 | *Brachionus quadridentatus melheni* |  |  |  |  |  |  |  | **+** |  |  | **+** |  |  |  | **+** | **+** |  |  | **+** |  | **+** |  |  | **+** |  |  |  |  |  |  |  |  |  |  |  |  |
| 50 | *Brachionus quadridentatus rhenanus* |  |  |  |  |  |  |  |  |  |  |  |  |  |  |  |  |  |  |  |  |  |  |  | **+** |  |  |  |  |  |  |  |  |  |  |  |  |
| 51 | *Keratella cochlearis* |  |  |  |  |  |  | **+** | **+** |  |  | **+** |  |  |  |  |  |  | **+** |  |  |  |  |  |  |  |  |  |  |  |  |  |  |  | **+** |  |  |
| 52 | *Keratella tecta* |  |  |  |  |  |  | **+** | **+** |  |  | **+** |  |  | **+** |  |  |  |  |  |  |  |  | **+** |  |  |  |  |  |  |  |  |  |  | **+** |  |  |
| 53 | *Keratella valga* |  |  |  |  |  | **+** | **+** | **+** |  |  |  |  |  |  |  |  |  |  |  |  |  |  |  | **+** |  |  | **+** |  |  |  |  |  |  |  |  |  |
| 54 | *Notholca labis* |  | **+** |  |  |  |  |  |  |  |  |  |  |  |  |  |  |  |  |  |  |  |  |  |  |  |  |  |  |  |  |  |  |  |  |  |  |
| 55 | *Platyias quadricornis* |  |  |  |  |  |  |  |  |  |  |  |  |  |  |  |  |  |  | **+** |  |  |  |  |  |  |  |  |  |  |  |  |  |  |  |  |  |
| 56 | *Colurella obtusa* |  |  |  |  |  |  |  |  |  |  |  |  |  |  |  |  |  |  |  |  |  |  | **+** |  |  |  |  |  |  |  |  |  |  |  |  |  |
| 57 | *Squatinella* sp. |  |  |  |  |  |  |  |  |  |  |  |  |  |  |  |  |  |  |  |  |  |  |  | **+** |  |  |  |  |  |  |  |  |  |  |  |  |
| 58 | *Lecane ungulata* |  |  |  |  |  |  |  |  |  |  |  |  |  |  |  |  |  |  |  |  |  |  |  | **+** |  |  |  |  |  |  |  |  |  |  |  |  |
| 59 | *Lecane luna* |  |  |  |  |  | **+** |  |  |  |  |  |  |  |  |  |  |  | **+** |  |  |  |  |  |  |  |  |  |  |  |  |  |  |  |  |  |  |
| 60 | *Monostyla bulla* |  |  |  |  |  |  |  |  |  |  |  |  |  |  |  |  |  | **+** |  |  |  |  | **+** | **+** |  |  |  |  |  |  |  |  |  |  |  |  |
| 61 | *Monostyla elachis* |  |  |  |  |  |  |  |  |  |  |  |  |  |  |  |  |  |  |  |  |  |  |  | **+** |  |  |  |  |  |  |  |  |  |  |  |  |
| 62 | *Euchlanis dilatata* | **+** | **+** |  |  |  | **+** |  |  | **+** |  | **+** | **+** |  |  |  |  |  |  |  | **+** |  |  | **+** |  |  |  |  | **+** |  |  |  | **+** | **+** |  | **+** |  |
| 63 | *Epiphanes senta* |  |  |  |  |  |  |  |  |  |  |  |  |  | **+** | **+** |  | **+** | **+** | **+** | **+** | **+** |  | **+** | **+** |  |  |  |  |  |  |  |  |  |  |  |  |
| 64 | *Asplanchna priodonta* |  |  |  | **+** |  |  | **+** | **+** | **+** |  |  | **+** |  |  |  |  |  |  |  |  |  |  |  |  |  |  |  |  |  |  |  |  |  |  | **+** |  |
| 65 | *Polyarthra dolichoptera* |  |  |  |  |  |  | **+** | **+** |  |  |  |  |  |  |  |  |  |  |  |  |  |  |  |  |  |  |  |  |  |  |  |  | **+** |  | **+** |  |
| 66 | *Polyarthra major* |  |  |  |  |  |  |  |  |  |  |  |  |  |  |  |  |  |  |  |  |  |  |  |  |  |  |  |  |  |  |  |  | **+** |  |  |  |
| 67 | *Polyarthra vulgaris* |  |  |  | **+** |  |  | **+** | **+** |  |  |  |  |  | **+** |  |  |  | **+** | **+** | **+** |  | **+** |  | **+** |  |  |  |  |  |  |  |  |  |  |  |  |
| 68 | *Synchaeta oblonga* |  |  |  |  |  |  |  |  |  |  |  |  |  |  |  |  |  | **+** | **+** |  |  | **+** | **+** | **+** |  |  |  |  |  |  |  |  |  |  | **+** |  |
| 69 | *Synchaeta pectinata* |  |  |  |  |  |  | **+** | **+** |  |  |  |  |  |  |  |  |  |  | **+** |  |  |  |  |  |  |  |  |  |  |  |  |  |  |  |  |  |
| 70 | *Synchaeta* sp. |  |  |  | **+** | **+** | **+** | **+** | **+** |  |  | **+** | **+** | **+** | **+** | **+** |  |  |  |  | **+** |  |  |  |  |  |  |  |  |  |  |  |  |  |  |  |  |
| 71 | *Cephalodella exigua* |  |  |  |  |  |  |  | **+** |  |  | **+** |  |  |  |  |  |  | **+** | **+** |  |  |  | **+** | **+** |  |  |  |  |  |  |  |  |  |  |  | **+** |
| 72 | *Cephalodella gibba* |  |  |  |  |  |  |  |  |  |  |  |  |  |  |  |  |  | **+** | **+** |  |  |  | **+** | **+** |  |  |  |  |  |  |  |  |  |  |  |  |
| 73 | *Encentrum* sp. |  | **+** |  |  |  |  |  |  |  |  |  |  |  |  | **+** | **+** | **+** | **+** | **+** | **+** |  | **+** | **+** | **+** |  |  |  |  |  |  |  |  |  |  |  |  |
| 74 | *Trichocerca rousseleti* | **+** |  |  |  |  | **+** |  | **+** | **+** |  | **+** |  |  | **+** |  |  |  |  |  | **+** |  |  |  |  | **+** | **+** |  |  |  |  |  |  |  |  |  |  |
| 75 | *Testudinella patina* |  |  |  |  |  |  |  |  |  |  |  |  |  |  |  |  |  |  |  |  |  |  |  |  |  |  |  |  |  |  |  | **+** |  |  |  |  |
| 76 | *Pompholyx complanta* |  |  |  |  |  |  |  | **+** |  |  |  |  |  |  |  |  |  |  |  |  |  |  |  |  |  |  |  |  |  |  |  |  |  |  |  |  |
| 77 | *Filinia cornuta* |  |  |  |  |  | **+** | **+** | **+** |  |  |  |  |  |  |  |  |  |  | **+** | **+** |  |  | **+** |  |  |  |  |  |  |  |  |  |  |  |  |  |
| 78 | *Filinia longiseta* |  |  |  |  |  |  | **+** | **+** |  |  |  |  |  |  |  |  |  |  | **+** | **+** |  |  | **+** |  |  |  |  |  |  |  |  | **+** | **+** |  | **+** | **+** |
| Cladocerans | | | | | | | | | | | | | | | | | | | | | | | | | | | | | | | | | | | | | |
| 79 | *Daphnia* *cucullata* | **+** |  | **+** |  | **+** |  |  |  |  |  |  |  |  |  |  |  |  |  |  |  |  |  |  |  |  |  |  |  |  |  |  |  |  |  |  |  |
| 80 | *Simocephalus vetulus* |  |  |  |  |  | **+** |  |  |  | **+** |  |  |  |  |  |  |  | **+** |  |  |  |  |  |  |  |  |  |  |  |  |  |  |  |  |  |  |
| 81 | *Scapholeberis mucronata* |  |  |  |  | **+** |  |  |  |  |  |  |  |  |  |  |  |  |  |  |  |  |  |  |  |  |  |  |  |  |  |  | **+** |  |  |  |  |
| 82 | *Moina micrura* |  |  |  |  |  |  |  |  |  | **+** | **+** |  |  |  |  |  |  |  |  |  |  |  |  |  |  |  |  |  |  |  |  |  |  |  |  |  |
| 83 | *Moina macrocopa* |  |  |  |  |  |  |  |  | **+** |  |  |  |  |  |  |  |  |  |  |  |  |  |  |  |  |  |  |  |  |  |  |  |  |  |  |  |
| 84 | *Bosmina coregoni* |  |  |  |  | **+** |  |  |  |  |  |  |  | **+** | **+** | **+** | **+** | **+** | **+** | **+** |  | **+** |  | **+** |  |  |  |  |  |  |  |  | **+** |  |  |  |  |
| 85 | *Bosmina fatalis* |  |  |  |  |  |  | **+** | **+** |  |  |  |  |  |  |  |  |  |  |  |  |  |  |  |  |  |  |  |  |  |  |  |  |  |  |  |  |
| 86 | *Bosminopsis deitersi* |  |  |  |  |  |  | **+** |  |  |  |  |  |  |  |  |  |  |  |  |  |  |  |  |  |  |  |  |  |  |  |  |  |  |  |  |  |
| 87 | *Ilyocryptus agilis* |  |  |  |  |  |  |  |  | **+** |  |  |  |  |  |  |  |  |  |  |  |  |  |  |  |  |  |  |  |  |  |  |  |  |  |  |  |
| 88 | *Ilyocryptus sordidus* |  |  |  |  |  |  |  |  |  |  |  |  |  |  |  |  |  |  |  |  |  |  |  |  |  |  |  |  |  |  |  |  |  |  |  | **+** |
| 89 | *Macrothrix rosea* |  |  |  |  |  |  |  |  |  |  |  |  |  |  |  |  |  |  | **+** |  |  | **+** |  |  |  |  |  |  |  |  |  |  |  |  |  |  |
| 90 | *Disparalona rostrata* |  |  |  |  |  |  |  |  | **+** |  |  |  |  |  |  |  |  |  |  |  |  |  |  |  |  |  |  |  |  |  |  |  |  |  |  |  |
| 91 | *Pleuroxus brevicornis* |  |  |  |  |  |  |  |  |  | **+** |  | **+** |  |  |  |  |  |  |  |  |  |  |  |  |  |  |  |  |  |  |  |  |  |  |  |  |
| 92 | *Pleuroxus trigonellus* |  |  |  |  |  |  |  |  | **+** |  |  |  |  |  |  |  |  |  |  |  |  |  |  |  |  |  |  |  |  |  |  |  |  |  |  |  |
| 93 | *Chydorus sphaericus* |  |  |  |  |  |  |  | **+** |  |  |  | **+** |  |  | **+** | **+** | **+** |  |  |  |  |  |  |  |  |  |  |  |  |  |  | **+** |  |  |  |  |
| 94 | *Camptocercus australis* |  |  |  |  |  |  |  | **+** |  |  |  |  |  |  |  |  |  |  | **+** |  |  |  |  |  |  |  |  |  |  |  |  | **+** |  |  |  |  |
| 95 | *Leydigia leydigii* |  |  |  |  |  |  |  |  | **+** | **+** |  |  |  |  |  |  |  |  |  |  |  |  |  |  |  |  |  |  |  |  |  | **+** |  |  |  |  |
| 96 | *Coronatella rectangula* |  | **+** |  |  |  |  | **+** |  |  |  |  |  |  |  |  |  |  |  | **+** |  |  |  |  |  |  |  |  | **+** |  |  |  | **+** | **+** | **+** | **+** |  |
| Copepods | | | | | | | | | | | | | | | | | | | | | | | | | | | | | | | | | | | | | |
| 97 | *Sinocalanus dorrii* | **+** | **+** | **+** |  | **+** |  |  | **+** |  |  |  |  | **+** | **+** | **+** |  |  |  |  |  |  |  | **+** | **+** | **+** | **+** |  | **+** |  |  |  |  |  | **+** |  |  |
| 98 | *Schmackeria forbesi* |  |  |  |  |  |  |  |  |  |  |  |  | **+** | **+** | **+** |  |  |  |  |  |  |  |  |  |  |  |  |  |  |  |  | **+** |  |  |  |  |
| 99 | *Neodiaptomus schmackeri* |  |  |  |  | **+** |  |  |  |  |  |  |  |  |  |  |  |  |  | **+** |  |  |  | **+** |  |  |  |  |  |  |  |  |  |  |  |  |  |
| 100 | *Macrocyclops albidus* |  |  |  |  |  |  |  |  |  |  |  |  |  |  |  |  |  |  |  |  |  |  |  |  |  |  |  |  |  |  |  |  |  |  |  | **+** |
| 101 | *Eucyclops serrulatus* |  |  |  |  | **+** | **+** |  |  | **+** | **+** |  | **+** |  |  |  |  |  |  |  |  |  |  |  |  |  |  |  |  |  |  |  |  |  |  |  |  |
| 102 | *Paracyclops fimbriatus* |  |  |  |  |  |  |  |  |  |  |  |  |  |  |  |  |  |  |  |  |  |  |  |  |  |  | **+** |  |  |  |  |  |  |  |  |  |
| 103 | *Ectocyclops phaleratus* |  |  |  |  |  |  |  |  | **+** |  |  |  |  |  |  |  |  |  |  |  |  |  |  |  |  |  |  |  |  |  |  |  |  |  |  |  |
| 104 | *Cyclops vicinus* |  |  |  |  |  |  |  |  | **+** |  |  |  |  |  |  |  |  |  |  |  |  |  |  |  |  |  |  |  |  |  |  |  |  |  |  |  |
| 105 | *Microcyclops varicans* |  |  |  |  |  |  |  |  |  |  |  |  |  |  |  |  |  |  |  |  |  |  |  |  |  |  |  |  |  |  |  | **+** |  |  |  |  |
| 106 | Canaloida Copepodid | **+** | **+** | **+** |  | **+** |  |  | **+** |  | **+** |  | **+** | **+** | **+** | **+** |  |  |  | **+** |  |  | **+** | **+** |  | **+** |  |  |  |  |  |  | **+** |  |  |  |  |
| 107 | Cyclopoida Copepodid | **+** |  | **+** |  | **+** | **+** |  | **+** | **+** | **+** | **+** |  | **+** | **+** | **+** | **+** | **+** | **+** | **+** | **+** | **+** | **+** |  | **+** |  |  |  |  |  |  |  | **+** | **+** |  | **+** | **+** |
| 108 | Nauplius | **+** | **+** | **+** |  | **+** |  |  | **+** | **+** | **+** | **+** |  | **+** |  | **+** | **+** | **+** | **+** |  | **+** | **+** |  |  |  | **+** | **+** | **+** | **+** |  |  |  | **+** |  |  | **+** | **+** |
