# Supplementary material for: Tempo-spatial variations of zooplankton communities in relation to environmental factors and the ecological implications: A case study in the hinterland of the Three Gorges Reservoir area, China
Source: PLoS One. 2021 Aug 18;16(8):e0256313. doi: 10.1371/journal.pone.0256313 (PMC8372925; doi:10.1371/journal.pone.0256313)
Supplement: Attachment — Submitted filename: Response to Reviewers.docx [file pone.0256313.s002.docx]

Journal Requirements:

When submitting your revision, we need you to address these additional requirements.

1. Please ensure that your manuscript meets PLOS ONE's style requirements, including those for file naming. The PLOS ONE style templates can be found at https://journals.plos.org/plosone/s/file?id=wjVg/PLOSOne_formatting_sample_main_body.pdf and https://journals.plos.org/plosone/s/file?id=ba62/PLOSOne_formatting_sample_title_authors_affiliations.pdf

Answer: Yes, we have followed the templates to revise the style of the manuscript and checked it again.

2. In your Methods section, please provide additional location information of the sampling sites, including geographic coordinates for the data set if available."

Answer: In fact, we have provided the geographic coordinates of all sampling sites in Fig 1. We add more detailed information in line 115-117.

3. In your Methods section, please provide additional information regarding the permits you obtained for the work. Please ensure you have included the full name of the authority that approved the field site access and, if no permits were required, a brief statement explaining why.

Answer: Actually, we are pretty sure that there is no need to declare the permits of the field investigations in the kind of public area in China. The local governments will not prevent this investigations, in contrast, they quite welcome it because this kind of work can provide the status of the water quality and biocenosis, being helpful to the ecological managements. And our fundings from Chinese government are completely open, not classified type, thus the local governments tend to support this field investigation. It can be said that that this type of field research papers where the study areas is located in China don’t need to provide the permits for their work.

4. PLOS requires an ORCID iD for the corresponding author in Editorial Manager on papers submitted after December 6th, 2016. Please ensure that you have an ORCID iD and that it is validated in Editorial Manager. To do this, go to ‘Update my Information’ (in the upper left-hand corner of the main menu), and click on the Fetch/Validate link next to the ORCID field. This will take you to the ORCID site and allow you to create a new iD or authenticate a pre-existing iD in Editorial Manager. Please see the following video for instructions on linking an ORCID iD to your Editorial Manager account: https://www.youtube.com/watch?v=_xcclfuvtxQ

Answer: Done

5. Thank you for stating the following in the Competing Interests section:

"The authors have declared that no competing interests exist."

We note that one or more of the authors are employed by a commercial company: "Jiangsu Environmental Engineering Technology Co. LTD"

a) Please provide an amended Funding Statement declaring this commercial affiliation, as well as a statement regarding the Role of Funders in your study. If the funding organization did not play a role in the study design, data collection and analysis, decision to publish, or preparation of the manuscript and only provided financial support in the form of authors' salaries and/or research materials, please review your statements relating to the author contributions, and ensure you have specifically and accurately indicated the role(s) that these authors had in your study. You can update author roles in the Author Contributions section of the online submission form.

Please also include the following statement within your amended Funding Statement.

“The funder provided support in the form of salaries for authors [insert relevant initials], but did not have any additional role in the study design, data collection and analysis, decision to publish, or preparation of the manuscript. The specific roles of these authors are articulated in the ‘author contributions’ section.”

If your commercial affiliation did play a role in your study, please state and explain this role within your updated Funding Statement.

Answer: Done as required, see the amended Funding Statement in the cover letter.

b) Please also provide an updated Competing Interests Statement declaring this commercial affiliation along with any other relevant declarations relating to employment, consultancy, patents, products in development, or marketed products, etc.

Within your Competing Interests Statement, please confirm that this commercial affiliation does not alter your adherence to all PLOS ONE policies on sharing data and materials by including the following statement: "This does not alter our adherence to PLOS ONE policies on sharing data and materials.” (as detailed online in our guide for authors http://journals.plos.org/plosone/s/competing-interests). If this adherence statement is not accurate and there are restrictions on sharing of data and/or materials, please state these. Please note that we cannot proceed with consideration of your article until this information has been declared.

Please include both an updated Funding Statement and Competing Interests Statement in your cover letter. We will change the online submission form on your behalf.

Please know it is PLOS ONE policy for corresponding authors to declare, on behalf of all authors, all potential competing interests for the purposes of transparency. PLOS defines a competing interest as anything that interferes with, or could reasonably be perceived as interfering with, the full and objective presentation, peer review, editorial decision-making, or publication of research or non-research articles submitted to one of the journals. Competing interests can be financial or non-financial, professional, or personal. Competing interests can arise in relationship to an organization or another person. Please follow this link to our website for more details on competing interests: http://journals.plos.org/plosone/s/competing-interests

Answer: Done as required, see the amended Funding Statement in the cover letter.

Additional Editor Comments (if provided):

This contribution analyses the water pollution status in urban aquatic ecosystem of Wanzhou City, spatio-temporal distribution patterns of zooplankton and investigated the associated ecological successions and key environmental factors shaping the community structures. The manuscript is acceptably written and analysed the data in appropriate ways. Following Reviewer 1,

I have the following comments to improve the quality of manuscript:

i) In abstract the authors can include quantified data when summarising salient observations.

Answer: Done, see line 14-34.

ii) Detailed methodologies of some of the major parameters like Chl-a can be explained in materials and methods.

Answer: Done, see line 135-137.

iii) For nutrient analysis, details on QA/QC procedures, calibration, detection limits can be given.

Answer: Done, see line 137-143.

iv) The authors need to be explained briefly CCA results on the manuscript.

Answer: Done. We further modified part context of the CCA results as suggested, see line 268-305.

v) Conclusion part need to be restructured and main results.

Answer: Done, see line 450-485.

vi) ‘n’ values need to be provided for Fig. 9.

Answer: Done.

Vii) The reported literature need to include the recent findings from 2020 and 2021.

Answer: Done.
